# Underfed Children

**Published:** 1905-05-06

**Authors:** 


### Underfed Children.

The Local Government Board has at last taken a very important step for the purpose of putting an end to the scandal concerning the attendance at school of underfed children, and has directed that Boards of Guardians, when they receive an application on the subject with reference to the case of any child under the age of sixteen, living with its father who is not in receipt of parish relief, shall not only take prompt steps to insure that the necessary food is supplied, but shall also investigate the case, and, if it be found that the underfeeding is due to parental neglect, shall take proceedings in the County Court to recover from the father the amount expended. The Court, in such a case, will have power to direct the father's employer to make any necessary deduction from wages, and to pay over the amount to the public officer appointed. Boards of Guardians are only to recognise applications made by an official appointed by the local education authority for the purpose, and they are to be at liberty either to supply the required food in the manner indicated above, as what is to be described as a "loan" to the father, or to give it as parish relief in the ordinary sense; exercising their discretion upon the facts as these are made known to them. The results of this experiment, for as such it must surely at present be described, will be watched with much interest by the public; and it will not be surprising if the application of the new order should be attended by unexpected difficulties of various kinds. There are probably many feeble children whose debility is not due so much to underfeeding as to disease or constitutional causes; and it is manifest that great care will have to be taken in order not to confound the two classes with one another. If the order is to work well, its operation must be so controlled as to avoid the production of "hard cases," which not only make bad law, but also may often excite public sympathy for entirely undeserving people. The discretion of the Guardians will also have to be exercised for the avoidance of proceedings in which the defendants would not even pay the costs, or possess any property which could be made available for the purpose. An important reason for the surrender of school pence was furnished by the frequent difficulty of obtaining them; and it is only to be expected that judgments for the repayments of loans would in many cases be totally inoperative, and would be nothing but sources of additional cost to the ratepayers.

Notwithstanding these difficulties, which it would be impossible to overlook, the fact that something is actually to be attempted for the purpose of putting an end to a very crying scandal is a matter for earnest congratulation. Although Mr. Gerald Balfour is the actual President of the Local Government Board, the new order has been issued too soon after his appointment to be entirely attributable to his initiative; and we shall probably not be wrong in regarding it as a fresh evidence of the practical spirit, and the determination to get to the root of things, of which Mr. Long has previously furnished some remarkable examples. Mr. Long's examination of the problem offered to the metropolis every winter by the necessities of the unemployed has rendered him familiar with the facts of poverty, and he is probably better acquainted than almost any other person with the relative proportions of genuine poverty and of neglect as causes of the underfeeding of children. It may be assumed, from the terms of the order, that neglect is at least so large an element in the case as to justify the belief that a considerable number of parents will be compelled by the new regulations to take more care of their children than they have done before, and it is probable that in this instance, as in so many others, law will become a powerful agency for the education of evil doers concerning the importance of duties which they have hitherto been accustomed to leave undone. However this may be, it is clear that for the future the presence of underfed children, who are more likely to suffer from schooling than to benefit by it, will only be rendered possible by the neglect of the school authorities themselves.

